# Biodiversity and Structural Analysis of Woody Plant Species of Home Gardens in Basona Worana District, North Shoa Zone of Central Ethiopia

**DOI:** 10.1155/2024/5563636

**Published:** 2024-02-08

**Authors:** Almaz Woldeyohannes, Admasu Moges

**Affiliations:** Department of Biology, Debre Berhan University, P.O. Box 445, Debre Berhan, Ethiopia

## Abstract

The purpose of this study was to investigate the biodiversity and structure of woody plants of HGs in the Basona Worana district (BWD). For this, six kebeles and 138 HGs from three agroecologies of BWD were selected using various sampling techniques. The plot size used per HG was 400 m^2^. Diversity and important value indices (IVIs) were computed. For their structural analysis, the diameter at breast height (DBH) and height were also measured for trees and shrubs fulfilling the standards (diameters at breast height (DBH) ≥2.5 cm and height >2 m). Thus, a total of 42 woody species belonging to 37 genera and 26 families were identified. Fabaceae was the most abundant family, followed by Euphorbiaceae. Trees were the dominant habit. The richness in Kola HGs (33) was higher than Dega (14) and Woinadega (19) HGs, with an overall mean richness of 4.14 per HG of BWD. The Shannon, Simpson, and evenness indices for HGs in the district were 1.05, 0.55, and 0.75, respectively, showing their moderate diversity with even distribution. The Sorenson's similarity of HGs between Dega and Woinadega, Kola and Dega, and Kola and Woinadega were 40%, 28%, and 32%, respectively. The multiple site similarities of woody species among three ecological zones (36%) were still <50%, showing no similarity. The overall DBH and height classes' patterns of the woody species individuals abruptly decreased towards their highest classes. Similar DBH and height classes' patterns of woody species individuals were also observed in Kola and Woinadega agroecologies, indicating their selective tree cutting. The mean values of DBH, height, basal, and crown areas of the woody species in the district were ∼14 cm, ∼6 m, ∼29 m^2^·ha^−1^, and 5 m^2^, respectively. Based on IVI, most of the HGs were dominated by *Eucalyptus* globules (93.35), followed by *Eucalyptus camaldulensis* (45.04), *Rhamnus prinoides* (22.4), and *Cupressus lusitanica* (22.33). Hence, actions should be taken to promote the diversity and managing of HGs' woody species of BWD.

## 1. Introduction

Biodiversity refers to the number, variety, and variability of living organisms; the different forms of life that exists in a given ecosystem, including the diversity within a species. Plant biodiversity is a subset of biodiversity, and it refers to the variety and variability of living plants in an area which can also be affected by the biotic and abiotic factors in that ecosystem [1].

Humans have been using plant resources to survive and to gain huge economic rewards since their existence on earth. Given plants play vital roles in providing food, medicine, energy, and wood for many uses such as furniture, building, and cultural materials. However, these plants' biodiversity undergoes chronic flattening changes. Huge hectares of forests are destroyed each year due to undue natural and manmade invasions, plant species diversity is fast fading, and many plant species have been dying out across the globe [2, 3].

Ethiopia has a wide range of ecological conditions ranging from the arid lowlands in the east to wet forests in the southwest and high altitude in the central highlands. This wide range of ecological conditions coupled with the corresponding cultural diversities has made the country to be one of the worldwide renowned major centers for biodiversity [4]. The Ethiopian flora is very diverse and the country owns above 6000 species of higher plants of which about 10% are endemic [5–7]. Regardless of this fact, the Ethiopian flora diversity has been facing complicated and severe threats [4].

Subsequently, the high forest cover of 16% of the total land area of Ethiopia, in the 1950s, has declined to below 3% during recent years [8]. Particularly, in the northern and northeastern parts of the country, the forest loss is huge. Because of the destruction of forests, many woody plants species have been cleared from their natural habitat, more than a few of them including the endemic woody species have become threatened, most of the mountainous sides have become naked sagging valleys, and streams that were used to have water over the whole years have now become dry in most of the seasons [7–9].

Thus, the conservation of natural flora diversity is known to have multifaceted uses, and it has been also identified to be the priority action to ensure the conservation of other natural resources [9]. Accordingly, countries around the globe have given notable attention to conserving their natural flora diversity [10]. Similarly, Ethiopia has started to conserve its natural flora diversity [4, 8].

Conservation of flora diversity can be achieved either by the protection of plant species in natural forests or by expanding the practice of HGs agroforestry systems [6, 11, 12]. The HG agroforestry system is a practice of management of multiuse trees and shrubs in closer association with annual and perennial crops and livestock in the backyards of each house, often using family labor [11]. The HG agroforestry system is potentially significant to reduce habitat fragmentation and increasing biodiversity in the agricultural scenery [12].

In Ethiopia, a nationwide tree-planting project was conceived as a major strategy to meet the needs of the local people and conserve the natural flora diversity during the 1970s [13]. Such coordinated tree-planting practices became declined and gradually vanished soon after a few years [6]. After that, HG agroforestry systems to protect woody species are recommended and taken as an option [7]. At the same time, it is strongly suggested that mobilizing the community nationwide and expanding HG agroforestry systems for conserving woody plant species is vital [4, 9]. However, such coordinated practice of HG agroforestry systems in Ethiopia stays inconsistent across various localities, and little attention has been given to taking advantage of the system [14, 15].

Several botanical studies relating to HGs have also been conducted in Ethiopia so far [9, 14, 16–18]. However, these and other previous studies in Ethiopia are merely confined to a few localities of the country, evidencing the lack of botanical information on woody plant species of HGs in most parts of the country. Because of that, conducting botanical studies to characterize woody plants species diversity, composition, and structure of HGs in various parts of Ethiopia, particularly in those areas which were not studied before, is vital. Moreover, to the best knowledge of the present researchers, the woody plant composition, diversity, and structure of the HGs in the present study area were not yet studied. Therefore, the main objective of this study was to determine the biodiversity and structure of woody plants of HGs in the Basona Worana district (BWD) of North Shoa zone, Ethiopia.

## 2. Materials and Methods

### 2.1. Description of the Study Area

#### 2.1.1. Location

**Figure 1:**
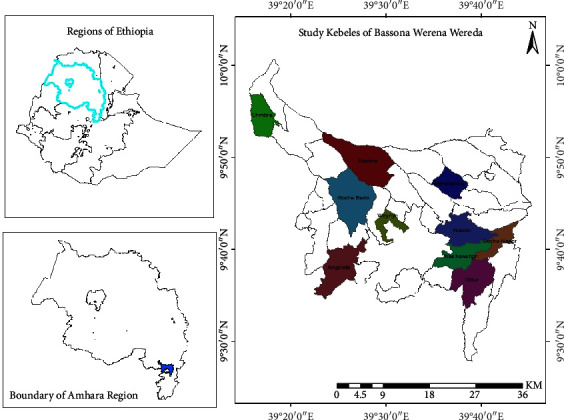
A map of the study area, where the highlighted 10 kebeles with different colors in Basona Worena Woreda were the study sites of the research work.

This study was conducted in the BWD of North Shoa zone, Amhara Regional state, Central Ethiopia. BWD is located surrounding the North Shoa zone's capital, called Debre Birhan town. It is found at 130 kilometers to the northeast of the capital city of the country, Addis Ababa, and at 695 km far from the Region's capital, Bahir-Dar city. In addition to the rural kebeles (where kebele is the smallest governmental administrative unit at the district or country level) of Debre Birhan town, the district is a neighbor of other districts, such as Tarmaber and Mojana Wodera in the north, Angolelana-Tera in the south, Ankober in the east, and Siyadebirna-Wayu, Moretina-Jiru, and Abichuna-Gne'a in the west. According to the information obtained from the Basona Worana Administration Office [19], the district covers a total area of 1, 208.17 km^2^ and is subdivided into 31 small administrative units called kebeles. Among the 31 kebeles, only one kebele is an urban kebele, while others are rural ones [19]. The map of BWD with the selected study kebeles is presented herewith (Figure 1).

#### 2.1.2. Population

According to the most recent population projection estimation [20] for 2014–2017, BWD has a total population of 140,386, of whom 71439 are males and 68947 females. Below 2% of the total population (2122), 1019 males and 1103 females are urban residents, while above 98% of the total population (138264), 70420 males and 67844 females are rural residents.

#### 2.1.3. Climate

**Figure 2:**
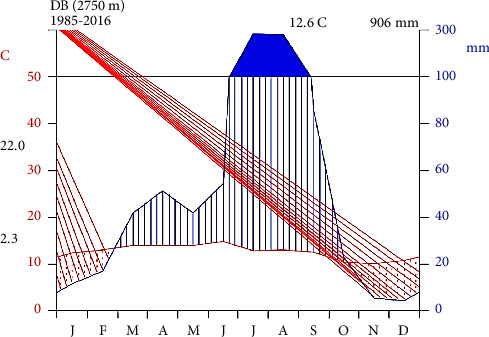
Climatic map of BWD [3].

The mean annual temperature of BWD ranges from 2.3°C to 22°C, and the annual rainfall ranges from 850 to 1100 mm with mean annual precipitation of about 906 mm per year (Figure 1). The distribution of rainfall in the area is unimodal, characterized by a prolonged wet season from June to September with the highest peak in July and August, while there is also a short rain falling between January and April (Figure 2). The daily temperature becomes very low from October to December when it may drop below zero, starts rising from January onwards, and gets to maximum in May (https://en.m.wikipedia.org/wiki/BasonaWerana). The information obtained from Basona Worana Agriculture and Rural Development Office (BWARDO) showed that most of the kebeles of the district are documented as highlands whilst only some are in lowlands; thus, about 90% of the total kebeles in the district receive a mean annual rainfall of 850–1000 mm [19]. On the whole, regarding their climatic conditions, 31 kebeles in the district are categorized into three agroecological zones, namely, Dega, Woinadega, and Kola [21].

#### 2.1.4. Topography

The topography of BWD is generally characterized by wider plains, undulating terrain, plateaus, mountains, hills, and fragmented valleys that are situated at an altitude ranging from 1500 to 3400 meters above the sea level (m.a.s.l). Given plains are estimated to cover about 70% and mountains cover 7% of the area's topography, while the remaining ones are valleys and cliffs.

### 2.2. Study Design

A reconnaissance survey was conducted from 5 to 20 January 2022 to introduce ourselves to local administrative bodies for getting information about the list of households, the availability of HG agroforestry practices, and their agroecological zones in BWD. The plant data collection was carried out from March 2022 to April 2022. Since the sampling sites (kebeles) were selected based on their agroecological zones (Dega, Woinadega, and Kola), a stratified random sampling design was applied. For the plant survey, a quadrat method per HG sampling design was applied, while for HG or household selection, a systematic random sampling design was applied by taking the list of the households from each selected Kebele Administrative Office.

### 2.3. Sample Size Determination

The sample size (the number of sample HGs) for this study was determined using the next single population proportion statistical formula [22] and assumptions of the proportion of households owing HGs [17].

(1) $$\begin{array}{c} n=\frac{Z_{\alpha /2}^{2}\text{pq}}{d^{2}}, \end{array}$$

where *n* is the desired sample size; *z* is the standard normal deviation at 95% confidence level (1.96), *p* is the proportion of households owing HGs (90%), and *d* is the degree of accuracy (0.05).

In that sense, **n**=(1.96)^2^ *∗* 0.9 (1 − 0.9)/(0.05)^2^=138. Therefore, 138 households (HGs) were the best possible sample sizes for this study. It is commonly agreed that a single HG is often too insufficient to take beyond a single study plot of adequate size (area) [9, 16–18], Jegora et al., 2019. Hence, the size (area) of each study plot was determined based on the size of study plots agreed to be adequate by similar previous studies conducted in Ethiopia [9, 16]. Accordingly, a total of 138 study plots (i.e., one plot per HG), each with an area of 400 m^2^ (i.e., 20 m × 20 m) were used for trees and shrubs.

The next task was to determine the number of sample households to be included from each of the three agroecological zones of the district (i.e., Dega (D), Woinadega (W), and Kola (K)), and from each of the sample kebeles in each agroecological zone. Regarding this, it is agreed that each household is likely to own its HG. Based on this assumption, sample HGs (households) were allocated using the next simple proportional formulae.

(2) $$\begin{array}{c} \text{nD}=\frac{\text{ND}}{N}\text{xn}\\ =\frac{16002}{32389}\text{x}138\\ =68,\\ \text{nW}=\frac{\text{NW}}{N}\text{xn}\\ =\frac{10653}{32389}x138\\ =45,\text{and}\\ \text{nK}=\frac{\text{NK}}{N}\text{xn}\\ =\frac{5734}{32389}x138\\ =25, \end{array}$$

where *nD*, *nW*, and *nK* are the sample households included among the total households in each of the three agroecological zones of BWD; the Dega, Woinadega, and Kola (i.e., *ND*, *N*W, and *NK*), respectively.

Likewise, the numbers of sample households from each sample kebeles in each agroecological zones of the district were determined based on proportional allocation to size using the next formulae.

(3) $$\begin{array}{c} \text{nDi}=\frac{\text{NDi}}{\overset{\;5}{\underset{i=1}{\sum }}\text{NDi}}\text{x nD},\\ \text{nWi}=\frac{\text{NWi}}{\sum_{i=1}^{3}\text{NWi}}\text{xnW},\text{and}\\ \text{nKi}=\frac{\text{NKi}}{\sum_{i=1}^{2}\text{NKi}}\text{xnK}, \end{array}$$

where *N*D*i*, *N*W*i*, and *N*K*i* are total households; and *n*D*i*, *n*W*i*, and *n*K*i* are sample households to be taken from a randomly selected kebele-*i* in the Dega, Woinadega, and Kola agroecological zones of the district, respectively, as shown in Table 1. The number of kebeles allotted to each one of the three agroecological zones was also based on the proportional allocation of total kebeles to be sampled from all to total kebeles in the given agroecological zone.

### 2.4. Sampling Techniques and Procedures

A total of 31 kebeles are found in BWD. These kebeles cover a wide area, so including all of them in the sample was unfeasible due to time, budget, and resource constraints. Also, directly selecting the households (HGs) at the district level randomly may lead to erroneous results due to potential variation of woody plant species because of urban and rural, as well as agroecological zone variations in the district. Therefore, to get a representative sample, this study applied a stratified random sampling technique followed by a systematic random sampling technique.

In the selection process, conditions that could cause sampling biases such as variation in urban or rural locations and in agroecological zones were adjusted. Thus, the urban kebele was purposefully excluded. Then, to adjust for variations in agroecological zones, the kebeles were stratified into Dega, Woinadega, and Kola agroecological zones, with 14, 10, and 6 rural kebeles in each, respectively. Among these 30 rural kebeles, ten kebeles comprising about 33.3% of the total were included and proportionally allotted to each agroecological zone. As a result, (14 *∗* 10)/30 = 4.7 ≈ 5 kebeles from Dega (i.e., Angolela, Bakelo, Dibut, Goshuager, and Wushawushign), (10 *∗* 10)/30 = 3.3 ≈ 3 kebeles from Woinadega (i.e., Goshe Bado, Mehal Amba, and Woiniye), and (6 *∗* 10)/30 = 2 kebeles from Kola (i.e., Kassima and Chimbre) were selected randomly (Table 1).

Finally, systematic random sampling was used to select the households (HGs). The first household (HG) in a given kebele was selected randomly, then the next household was the (1 + *k*)^th^ household, then the third (1 + 2*k*)^th^, the fourth (1 + 3*k*)^th^, etc., where, *k*, which is called a sampling interval, was calculated by dividing the total number of households in a given kebele by the number of sample households allotted for that kebele. The sampling interval (*k*) for each of the ten randomly selected kebeles is given in Table 1. At the same time, during the field survey, the list of households in each of the Rural Kebele Administration Offices was used as a sampling frame. If a household that did not have its own HG was selected by the systematic random selection method during the field survey, the data collector moved to the neighboring household (i.e., to the *n* + 1, *n* + 2, *n* + 3…etc.), until the next interval (i.e., *n* + *k*) was reached.

### 2.5. Plant Data Collection and the Identification Procedure

Botanical data were taken from a plot with a size of 20 m × 20 m per HG. Thus, all woody plant species in each plot were counted and recorded using their vernacular names with the aid of owners of HGs [9]. Besides, physiographic data such as altitude, latitude, and longitude were recorded for each HG using GPS, while the systematic classification was carried out following Tesemma [23] and Ermias [24].

In addition, to determine the woody species structure and dominant species, biometric parameters such as diameter at breast height (DBH) and height were taken for all the trees in each main plot (Supplementary file 1). In this study, trees/shrubs were defined as woody plants with diameters at breast height (DBH) ≥2.5 cm and height >2 m. Particularly, a tree was defined as a woody perennial plant with a single main stem or in the case of coppice several stems and has more or less a definite crown, while shrubs were defined as woody perennial plants, often lacking a definite crown, with several stems growing from the same root.

Furthermore, the canopy/crown cover (diameter) of each woody species was measured using a measuring tape (Supplementary File 1) for determining the species diversity of the HG [25]. Finally, all plant specimens collected were pressed, dried, and taken to the Department of Biology of Debre Berhan University for identification. The identifications of the plant specimens were carried out using the flora volumes of Ethiopia and Eritrea, besides being assisted by experts. Of course, further identification was made using mounted samples and a microscope in the Herbarium of Addis Ababa University and then the voucher specimens and their copies were deposited at Addis Ababa and Debre Birhan Universities, respectively.

### 2.6. Data Processing and Analysis

Data were entered, cleaned, and coded in an Excel spreadsheet, while further statistical analyses were performed using Stata Software with version 14.22. Descriptive statistics such as frequencies, percentages, and means of diameter at the breast height (DBH), height, and basal area were computed to describe woody species structures of the HGs. Besides, analysis of variance (ANOVA) was used to compute multiple comparisons of means.

Species richness (S), Shannon–Weiner diversity index (H), equitability/evenness (J), and species dominance using the Simpson dominance index (*D*) [26, 27], and important value index (IVI) were computed using the following approach/formulae.

Species richness (*S*) was calculated by using the following equation:

(4) $$\begin{array}{c} S=\sum \text{ni}, \end{array}$$

where *ni* is the number of species in a community.

The Shannon–Weiner diversity index (*H*′) was calculated using the following equation:

(5) $$\begin{array}{c} H^{\prime }=-\overset{S}{\underset{i=1}{\sum }}\text{Piln}\left(\text{Pi}\right), \end{array}$$

where *H*′ = Shannon diversity indices, *S* = number of species in the community, and Pi = proportion of individuals found in the i^th^ species (i.e., Si/N; where Si = number of individuals of the i^th^ species, *N* = total number of individuals of all the species).

Equitability/evenness (*J*) was calculated using the following equation:

(6) $$\begin{array}{c} J=\frac{H^{\prime }}{H\;\max}\\ =\frac{-\sum_{i=1}^{s}\text{Piln}\left(\text{Pi}\right)}{\ln\;S}, \end{array}$$

where *S* = number of species; *H*′ = Shannon diversity indices; and Pi = proportion of individuals found in the *i*^th^ species (i.e., *H*_max_ is equal to *lnS*).

The Simpson dominance index (*D*) was calculated using the following equation:

(7) $$\begin{array}{c} D=1-\overset{s}{\underset{i=1}{\sum }}{\text{Pi}}^{2}, \end{array}$$

where *D* = Simpson's index of species diversity; *S* = number of species; and Pi = Proportion of a total sample belonging to the *i*^th^ species.

The Sørensen similarity coefficient (SS) [28] was used to calculate the species similarities between HGs of in each pair of agroecological zones. The SS is defined by the following equation:

(8) $$\begin{array}{c} \text{SS}=\frac{2a}{\left(2a+b+c\right)}, \end{array}$$

where SS = Sørensen similarity coefficient, *a* = number of species common to both samples, *b* = number of species in the HGs of the “*b*” agroecological zone, and *c* = number of species in the HGs of the “*c*” agroecological zone.

The SS measure is limited to comparisons of similarities between a pair of sites at a time, while the multiple site similarity index (MSSI) is a method suggested to conquer the limitations of methods restricted to pairwise comparisons [29]. Thus, in addition to the aforesaid pairwise similarity measures, the overall similarity of woody species diversity among the three agroecological zones was analyzed using the MSSI using the following formula.

(9) $$\begin{array}{c} \text{MSSI}=\frac{\text{ab}+\text{ac}+\text{bc}-\text{abc}}{a+b+c}, \end{array}$$

where MSSI = multiple site similarity index, *a* = number of species found in the Dega agroecology, *b* = number of species found in the Woinadega agroecology, *c* = number of species found in the Kola agroecology, ab = number of species common to the Dega and Woinadega agroecologies, ac = number of species common to the Dega and Kola agroecologies, bc = number of species common to the Woinadega and Kola agroecologies, and abc = number of species found in the three agroecologies.

The crown area was calculated following Bajigo and Tadesse (2015). This means that

(10) $$\begin{array}{c} \text{Crown area}=\pi \;\left(0.5*{\left(\text{average crown diameter}\right)}^{2}\right). \end{array}$$

The important value index (IVI) is a composite index computed based on the relative density, relative dominance, and relative frequency. It shows the significance of species in a system [18]. In this study, IVI was computed for all woody plant species based on the next formulae:

(11) $$\begin{array}{c} \text{IVI}=\text{relative frequency}+\text{relative abundance}+\text{relative dominance}, \end{array}$$

where

(12) $$\begin{array}{c} \text{Relative Frequency}=\frac{\text{frequency of individual woody species}}{\text{ frequency of all woody species}}\;X\;100,\\ \text{Relative Dominance}=\frac{\text{basal area of individual woody species}}{\text{total basal area of all species }}\;X\;100,\\ \text{Relative Abundance}=\frac{\text{number of individuals of }a\text{ species}}{\text{ number of individuals of all woody species}}\;X\;100. \end{array}$$

## 3. Results

### 3.1. Home Gardens' Characterization

In this study, a total of 138 HGs found in ten rural kebeles of BWD were surveyed. The HGs were located in three agroecological zones of the district with altitudes ranging from 1606 to 3039 m.a.s.l. Of the total HGs, 68, 45, and 25 were found in Dega, Woinadega, and Kola agroclimatic zones, respectively (Table 2).

### 3.2. Trees and Shrub Species Composition and Their Growth Forms

**Figure 3:**
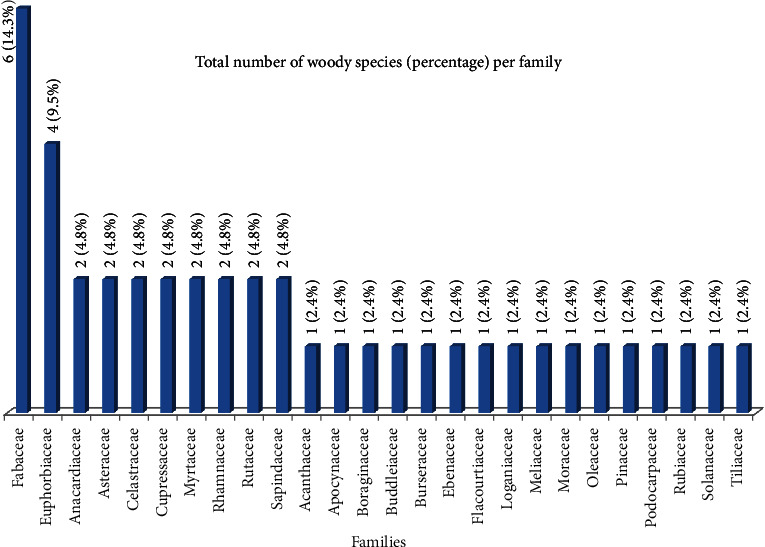
Distribution of the families with their total number of species per family in BWD.

**Figure 4:**
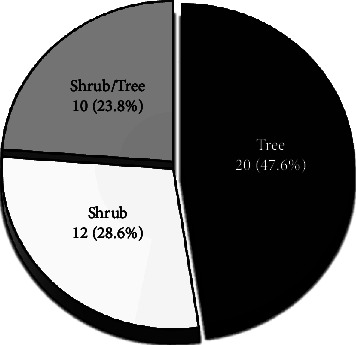
The growth forms of the woody plant species identified from HGs of the study area.

A total of 42 tree and shrub species belonging to 37 genera and 26 families were identified (Table 3). Of the total families, Fabaceae consisting of 6 species (14.3%) was the leading family followed by Euphorbiaceae with 4 species (9.5%) (Figure 3). However, the remaining eight and 16 families were represented by two and one species (each), respectively (Figure 3). Regarding the growth forms of the plant species, among 42 woody species, 20 species (47.6%) existed as trees only, 10 species (23.8%) as both shrubs and trees, while 12 (28.6%) of the woody species as shrubs only (Figure 4).

### 3.3. The Species Distribution across Agroecological Zones of the Study Area

**Figure 5:**
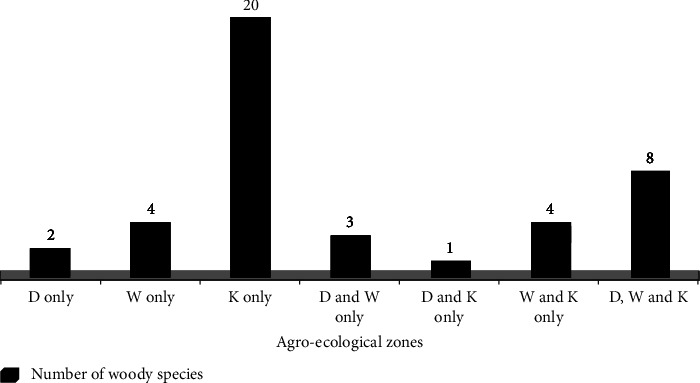
Distribution of woody species in agroecological zones of BWD (where *D* = Dega, *W* = Woinadega, and *K* = Kola).

As displayed in Figure 5 and Supplementary File 2, the distribution of tree and shrub species of the HGs in the three agroecological zones of the district was presented as follows. Two of the species, namely, *Ricinus communis* and *Acacia melanoxylon* were found only in the Dega agroecological zone. Four of the species including *Maytenus arbutifolia*, *Ekebergia capensis*, *Schinus molle,* and *Podocarpus falcatus* were found only in the Woinadega agroecological zone, whereas 20 of the species including *Commiphora africana*, *Catha edulis*, *Citrus sinensis,* and *Ficus sycomorus* were found only in the Kola agroecological zone. Furthermore, three species including *Cupressus lusitanica*, *Chamaecytisus proliferus,* and *Pinus patula* were found only in the Dega and Woinadega; one tree species of *Juniperus procera* was found only in the Dega and Kola; four species such as *Acacia abyssinica*, *Dodonaea viscosa*, *Croton macrostachyus,* and *Carissa spinarum* were found only in the Woinadega and Kola agroecological zones, while the remaining eight species such as *Erythrina abyssinica shrub*, *Eucalyptus camaldulensis,* and *Eucalyptus globulus* trees were found in all of the three agroecological zones (Figure 5).

### 3.4. Richness and Abundance of Woody Species

**Figure 6:**
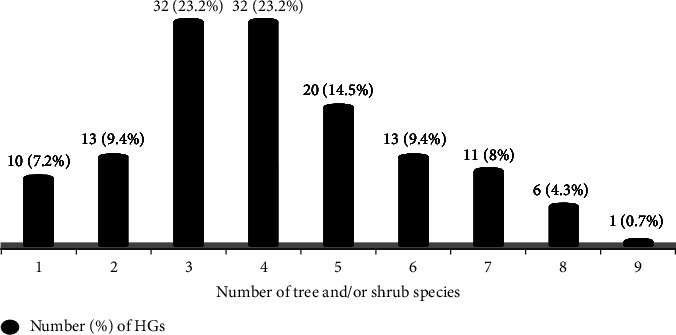
Number of tree and/or shrub species per plot in HGs of BWD.

The number of trees and/or shrub species found in each of the surveyed HGs ranges from a minimum of one to a maximum of nine woody species (Figure 6). Accordingly, among the total of 138 HGs, 10 (7.2%) of the HGs each had a single tree/shrub species; 13 (9.4%), only two woody species; 32 (23.2%), only three woody species; 32 (23.2%), only four woody species; 20 (14.5%), only five woody species; and 13 (9.4%), only six woody species; while the remaining 18 (13%) had more than six woody species in each (Figure 6). So, each of the HGs had approximately four species richness on average.

The trees and shrub species richness of the HGs concerning the locations in the three agroecological zones of BWD was also presented (Table 4). Accordingly, the total number of woody species (richness) of the HGs in the Dega agroecological zone was 14, 19 in Woinadega, and 33 in Kola. These results showed that the woody species richness of HGs in Kola, Woinadega, and Dega agroecological zones decreased from Kola to Dega ones (Table 4).

Likewise, the numbers of woody species about the HGs of each kebele were also analyzed. Based on this, the minimum total of eight woody species was recorded in the HGs of one of the five study kebeles located in the Dega agroecological zone called “Dibut”. Whilst the maximum total of 24 woody species were recorded in the HGs of one of the two studied kebeles located in the Kola agroecological zone of the district called “Chimbre” (Table 4).

Regarding the abundance of the species, a total of 5475 individuals belonging to the 42 woody species were counted in the surveyed HGs. Among the total, 3259 individuals belonged to the HGs of Dega, 1610 to Woinadega, and 606 to Kola agroecological zones. Accordingly, the abundance of woody species found in the HGs of Dega, Woinadega, and Kola agroecological zones ranked as first, second, and third, respectively. Similarly, the HGs in “Wushawushign”, “Bakelo,” and “Angolela” kebeles in the Dega agroecological zone were ranked as first, second, and third, respectively (Table 5).

### 3.5. Similarity Index Values of Woody Species

The comparisons were made between each pair of agroecological zones separately using SS, as well as among the three agroecological zones at the same time using MSSI (Table 6). Accordingly, based on the pairwise comparison of SS of the woody species composition, the highest SS (40%) was recorded between the HGs found in Dega and Woinadega agroecological zones; whereas the lowest SS (28%) was recorded between the HGs located in the Dega and Kola agroecological zones. At the same time, the overall MSSI measure of the woody species composition among the three agroecological zones was recorded as 36% (Table 6).

### 3.6. Multiple Comparisons and Statistical Tests for Diversity Indices across Three Agroecological Zones

The mean values of richness, abundance, and other diversity indices were compared for measuring the woody species diversity of the HGs across the three agroecological zones of BWD (Table 7). Accordingly, the mean richness of the woody species of the HGs across the Dega, Woinadega, and Kola agroecological zones were 3.21, 4.60, and 5.88, respectively, while, the overall mean richness of the woody species of the present study area was 4.14, indicating low richness. Likewise, the mean abundances of woody species of the HGs, located at Dega, Woinadega, and Kola agroecological zones were 47.93, 35.78, and 24.24, respectively; whilst the overall mean abundance of woody species for the HGs of the district was 39.67 (Table 7). The results showed that the mean rich values were increased from Dega to Kola but vice versa for mean abundance values.

The mean values of the Shannon diversity index (*H*′) of the HGs, situated at Dega, Woinadega, and Kola agroecological zones, and at the whole study area were 0.84, 1.14, 1.48, and 1.05, respectively. The mean values of the Simpson's diversity (*D*) for HGs across Dega, Woinadega, and Kola agroecological zones and the whole study area were 0.47, 0.60, 0.72, and 0.55, respectively. Likewise, the mean values of evenness (*H*′/*H*_max_) of woody species for HGs in the Dega, Woinadega, and Kola agroecological zones were 0.69, 0.78, and 0.86, respectively, whilst overall mean values of the HGs in the district as a whole was 0.75 (Table 7), indicating the increasing up of all values of diversity indices from Dega to Kola agroecological zones.

The analyses results of the multiple comparisons for mean values of the richness of woody species revealed the presence of statistically significant differences between the HGs in each pair of the three agroecological zones (i.e., between Dega and Woinadega, Dega and Kola, and Woinadega and Kola) at *p* values of less than (<) 0.05 (Table 8). Whilst the multiple comparisons for means of abundance of woody species revealed the presence of statistically significant difference only between the HGs situated at Dega and Kola agroecological zones (Table 8).

Still, the results of multiple comparisons for means of the Shannon diversity (*H*′) and Simpson's diversity index (*D*) of woody species revealed the presence of statistically significant differences between each pair of the three agroecologies at *p* values of <0.05 (Table 8), respectively. Contrarily, the comparisons for means of evenness (*H*′/*H*_max_) revealed the presence of significant differences only between the HGs in Dega and Kola agroecologies (Table 8).

### 3.7. Woody Species Community Structure of HGs

#### 3.7.1. DBH Classes Distributions of Woody Species

**Figure 7:**
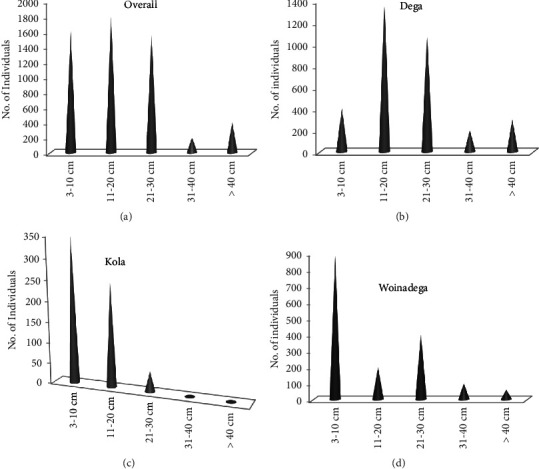
Diameter classes (cm) distribution of the woody species in HGs of BWD. (a–d) DBH categories.

**Figure 8:**
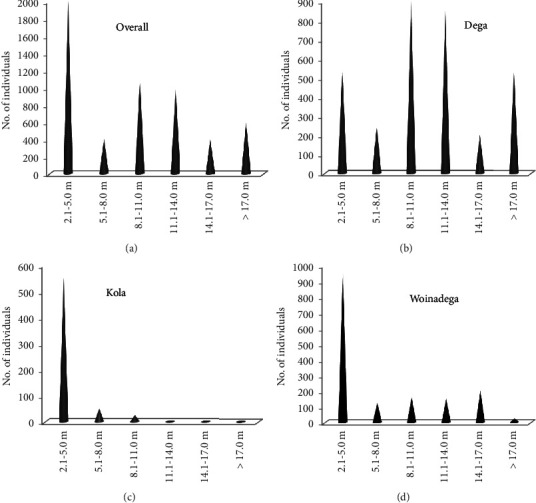
Height classes (m) distribution of the woody species in HGs of BWD. (a–d) Height categories.

The community structure was constructed based on the dimensions of DBH and height categories for the woody species of HGs of the district as a whole and of the three agroecological zones of the district (Figures 7 and 8). The five DBH classes of the woody species were class 1, 3−10 cm; class 2, 10.1−20 cm; class 3, 20.1−30 cm; class 4, 30.1−40 cm; and class 5, >40 cm. The results for DBH classes' pattern of the woody species of HGs in BWD as a whole showed that the number of individuals decreased abruptly as the dimension of DBH increased starting from DBH class 2 (Figure 7(a)). Yet, the results of DBH classes in the Dega (Figure 7(b)), Kola (Figure 7(c)), and Woinadega (Figure 7(d)) agroecologies of the district showed variations. For instance, most individuals of the woody species in HGs of the Dega agroecology were clustered at the second class, followed by the third class, but not at the first, fourth, and fifth classes (Figure 7(b)). Hence, the DBH class categories of the woody species of the HGs at Dega agroecology showed somewhat bell-shaped. The number of individuals in DBH class categories of the woody species at Kola agroecology showed regularly decreasing as the DBH of the individual increased starting from DBH class 1 to class 5, indicating an inverted J-shaped. While looking at the pattern of the individuals of the woody species, DBH classes of HGs situated at Woinadega were totally unlike the pattern of Dega but somewhat looked like the Kola agroecology. This means that the pattern of the individuals of the woody species in DBH classes of HGs located at the Woinadega agroecology was abruptly decreased towards the higher class 5, showing an abruptly inverted J-shaped (Figure 7(d)).

#### 3.7.2. Height Classes Distributions of Woody Species

The height classes of the woody species were also computed into the following 6 classes: class 1, 2.1–5 m; class 2, 5.1–8 m; class 3, 8.1−11 m; class 4, 11.1−14 m; class 5, 14.1−17 m, and class 6, >17 m. Hence, the results for height distribution of individuals of the woody species in the HGs of BWD as a whole showed that the number of individuals abruptly decreased as the height of the individual increased (Figure 8(a)). In this pattern, therefore, most individuals were clustered in the lowest class, followed by the third and fourth classes. The distribution of the woody species class patterns of HGs in Dega agroecology (Figure 8(b)) was, however, somewhat different to the whole study area but almost similar pattern to Kola (Figure 8(c)) and Woinadega (Figure 8(d)) agroecologies. This means that the height class patterns of woody species growing in Kola and Woinadega agroecologies abruptly decreased as passing from the lowest to the highest classes. Yet, most individuals of the woody species in HGs of Dega agroecology were almost clustered in the middle two classes, followed by the lowest and the highest classes (Figure 8(b)).

#### 3.7.3. Stand Characteristics of the Woody Species

Stand variables were computed in terms of means of DBH, height, basal area per hectare, and crown area for woody species in HGs of BWD (Table 9). The overall means of the respective stand characteristics were computed for the woody species in the HGs of the district as a whole as well as in each of the three agroecological zones.

The overall mean DBH of the woody species in the district was 14.23 cm, while the mean DBH for the woody species in the HGs of the Dega, Woinadega, and Kola agroecological zones was 17.3 cm, 12.92 cm, and 11.54 cm, respectively. The overall mean height of the woody species in the district was 6.04 m, while the mean height for the woody species in the HGs of the Dega, Woinadega, and Kola agroecological zones was 7.76 m, 5.3 m, and 4.54 m, respectively. The overall mean basal area for the woody species in the district was 29.02 m^2^ per hectare, while the mean basal area for the woody species in the HGs of the Dega, Woinadega, and Kola agroecological zones was 40.1, 24.86, and 6.5 m^2^/ha, respectively. Likewise, the overall mean crown area of individuals of the woody species (trees) in the district was 5 m^2^, while the mean crown area for the trees in the HGs of the Dega, Woinadega, and Kola agroecological zones was 4.92, 4.14, and 6.35 m^2^, respectively (Table 9).

#### 3.7.4. Statistical Test Analysis for Mean Values of Stand Variables

Multiple test comparisons based on the means of each of the respective stand variables of the woody species were made among the HGs found in the three agroecological zones (Table 10). Hence, there were strongly significant differences between the means of DBH of the woody species of Dega and Woinadega as well as Dega and Kola at *p* values <0.001. However, there was no significant difference in the mean differences of DBH between Woinadega and Kola agroecologies (Table 10).

Likewise, there were also strong significant differences between the means of height of the woody species in the HGs of Dega and Woinadega and Dega and Kola at *p* values <0.001 (Table 10). Yet, there was no significant difference between the means of the woody species of the HGs found in Woinadega and Kola agroecological zones (Table 10). Regarding the statistical test for means of the basal area of the woody species in the HGs, there was also a strongly significant difference between Dega and Kola agroecological zones (*p* value <0.001) but not among other agroecological zones (Table 10). In addition, there was not a significant difference in the means of crown areas of the trees of the HGs situated between Dega and Woinadega agroecologies. Yet, there were significant differences at *p* values of 0.018 and 0.001 for the mean differences of the crown areas of the trees found between Dega and Kola as well as between Woinadega and the Kola agroecologies, respectively (Table 10).

**Figure 9:**
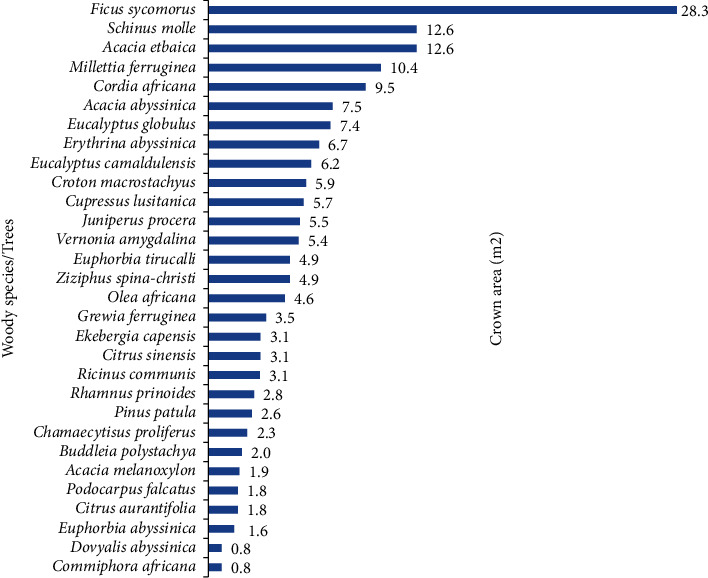
Crown areas of the woody species/trees in HGs of the study area.

The mean crown area for each of the woody species (trees) was also computed (Figure 9). Based on this, *F. sycomorus*, *S. molle, Acacia etbaica, Millettia ferruginea*, and *Cordia africana* were the top five wood plant species with their largest crown areas, respectively.

### 3.8. Importance Value Index (IVI)

The IVI was analyzed for each of the entire woody species (42) identified from the HGs of the district (Table 11). The results showed that *Eucalyptus globules* (93.5%), *Eucalyptus camaldulensis* (45.04%), *Rhamnus prinoides* (22.40%), *Cupressus lusitanica* (22.33%), *Croton macrostachyus* (16.94%), *Pinus patula* (13.17%)*, Buddleia polystachya* (12.44%)*, Acacia abyssinica* (9.32%)*, Juniperus procera* (9.23%)*, and Euphorbia abyssinica* (7.25%) were the top ten important woody species in the HGs of BWD (Table 11).

The IVI was analyzed among the woody species of the three agroecological zones of the district presented underneath (Table 12). Accordingly, *E. globules* (110.88%), *E. camaldulensis* (51.36%), *C*. *lusitanica* (32.09%), *P*. *patula* (24.73%), and *R*. *prinoides* (22.21%) were the top five woody species found in the Dega agroecological zone. *E. tirucalli (57.02%), C*. *macrostachyus* (43.47%), *C*. *africana* (28.59%), *R*. *prinoides* (27.65%), and *E*. *abyssinica* (27.56%) were the top five woody species in the Kola agroecological zone. *E. globulus* (87.06%), *E*. *camaldulensis* (47.22%), *C*. *macrostachyus* (33.38%), *R*. *prinoides* (23.95%), and *A*. *abyssinica* (21.25%) were the top five woody species found in Woinadega agroecological zone. These results showed regarding the important woody species that there were minor differences between Dega and Woinadega but more between Kola and Dega/Woinadega agroecological zones (Table 12).

## 4. Discussion

### 4.1. Woody Species Composition

In this study, a total of 42 woody species were identified in the HGs of BWD, while a total of 5475 matured individuals of all woody species were counted in the same area, which gave an overall mean of 39.67 individuals per HG. The present study finding regarding the total richness was equivalent to the finding of Mengistu and Asfaw [14], from Dallo Mena District Central Ethiopia. The similarities of the findings might be associated with the relative similarities in the topography of the study areas and in the farmers' practice to plant woody species in their HGs. Yet, the present study finding was higher than the number of woody species reported by the study of Eyasu et al. [9] conducted in Southern Tigray, Northern Ethiopia, but quite lower than the number of woody species reported by the study of Humnessa [18] carried out in Dandi District, Central Ethiopia. These disparities might also be explained by differences in topographies of the study areas, and in other socioeconomic and cultural variations, which could affect the farmers' preferences and practices of planting woody species in their HGs.

Regarding the growth forms, the majority of the woody species identified in HGs of BWD existed as trees only, while the woody species existed as shrubs only, and as shrubs and trees ranked second and third, respectively. This finding of the present study was consistent with the findings of the previous studies conducted in southern [16] and northern [9] Ethiopia.

The present study also revealed a total of 14, 19, and 33 woody species in HGs of the Dega, Woinadega, and Kola agroecological zones, which also gave a mean woody species richness of 3.21, 4.60, and 5.88 in HGs of the Dega, Woinadega, and Kola agroecological zones, respectively. Moreover, the total mature individuals (mean per HG) of woody species of the HGs found in Dega, Woinadega, and Kola agroecological zones were 3259 (47.93), 1610 (35.78), and 606 (24.24), respectively. Regarding overall woody species composition, the highest similarity existed between the HGs of the Dega and Woinadega agroecological zones, whereas the lowest similarity was recorded between the HGs of Dega and Kola agroecological zones. Generally, the similarity within the HGs in the three agroecological zones was quite lower than half, implying the similarity of plants growing in Kola, Woinadega, and Dega was very low, which was resulting from their agroclimatic and topographic conditions, besides farmers' experience differences. This finding was in agreement with a study conducted in Southern Ethiopia [16]. The variation in species richness and abundance of HGs among the three agroecological zones might be due to each agroecological zone best fits to hold diverse woody species. But, as opposed to the explanation given by a previous study, the variations were not just linked with the sum of HGs surveyed in the respective agroecological zone [18].

### 4.2. Woody Species Diversity

Based on the present study, the overall mean woody species richness of the HGs in the district was 4.14, showing that only a small number of woody species were available in each of the HGs in the district. This finding was comparable with the finding of a study conducted in Southern Ethiopia [16]. It was also quite higher than the finding of the study done in Bulen district, Northwestern Ethiopia [17]. However, this finding was lower than the findings of the studies conducted in Dandi district, Central Ethiopia [18] and Southern Tigray, Northern Ethiopia [9]. The finding of the present study also showed a statistically significant difference in the mean richness of woody species among the HGs in the three agroecological zones of the district. In addition, there was a statistically significant difference in mean woody species abundance between the HGs of the Dega and the Kola agroecological zones of the district. These findings were in agreement with the findings of a study conducted in Southern Ethiopia [16].

The mean values of Shannon diversity (*H*′) and Simpson's diversity (*D*) for HGs in the present study area were 1.05 and 0.55, respectively, indicating moderate diversity. These figures were also quite lower than the findings of the studies conducted in different parts of Ethiopia [9, 16–18]. There were also statistically significant differences for the mean values of the Shannon diversity (*H*′) and Simpson's diversity (*D*) among Dega, Woinadega, and Kola agroecological zones of the district, which were in agreement with the finding of Tefera et al. [16]. Still, the mean value of evenness of the species in the present study area (0.75) was in agreement with the findings of Eyasu et al. [9]; Tefera et al. [16]; Humnessa [18]; and Beyene et al. [17]. However, there was a statistically significant difference in the means of evenness only between the HGs located at the Dega and Kola agroecological zones.

### 4.3. Woody Species Community Structure of HGs

The overall results for the woody species structure in HGs of the district showed that the number of individuals decreased as the DBH and height of the individuals increased (i.e., the number of individuals of woody species abruptly decreased from the lower to higher DBH and height classes), implying the selective cutting of matured trees there. Likewise, although a big difference was seen in structural patterns of DBH and height classes for individuals of the woody species in HGs of between the overall study area and Dega agroecological zone, there were more similarities in DBH and height class patterns between the overall study area and Woinadega/Kola agroecological zones. The present findings were, therefore, in agreement with the results revealed by several studies conducted in different parts of Ethiopia [9, 16–18]. The mean values of DBH (∼14 cm), height (∼6 m), basal (∼29 m^2^·ha^−1^), and crown (5 m^2^) areas of the woody species in the district agreed with the findings of Tefera et al. [16] and indicated the existence of fair plant stand of HGs in BWD, which, in turn, pointed out the demand of the scientific managing of HGs.

### 4.4. Importance Value Index

The results of IVI of the present study revealed that *E*. *globulus* and *E*. *camaldulensis*, followed by *R*. *prinoides*, *C*. *lusitanica*, and *C*. *macrostachyus*, were the top five important woody species in HGs of the district. Moreover, *E. globulus*, *E*. *camaldulensis*, *C*. *lusitanica*, *P*. *patula*, and *R*. *prinoides* were the top five woody species in Dega agroecology. Still, *E*. *globulus*, *E*. *camaldulensis*, *C*. *macrostachyus*, *R*. *prinoides*, and *A*. *abyssinica* were the top five woody species in the Woinadega agroecological zone. Contrarily, *E*. *tirucalli, C*. *macrostachyus*, *C*. *africana*, *R*. *prinoides*, and *E*. *abyssinica* were the top five woody species in the Kola district. The latter results showed slight differences among the three agroecological zones regarding the important woody plant species. In all cases, regarding the important woody species in the HGs, the present study finding was quite different from the findings of previous studies [9, 16–18].

## 5. Conclusions and Recommendations

Forty-two woody plant species belonging to 26 families were identified in HGs of BWD, indicating the HGs can be good alternatives to natural forests for maintaining the woody species wealth of an area. Of the total (26), the family Fabaceae, followed by Euphorbiaceae, was dominant, which might be due to the farmers' interest and agroclimatic conditions. Most of the woody species have existed as trees only. The woody species richness of the HGs was significantly different among the three agroecological zones of the district, with the highest species richness in the HGs of the Kola agroecology. The woody species abundance in the HGs of the Dega was significantly higher than that in the Kola agroecological zone but not between the HGs of the Dega and Woinadega agroecological zones. Moreover, the overall woody species composition similarity among the HGs in BWD and across the three agroecological zones of the district was very low (below half) due to the differences in farmers' experiences and agroclimatic and topographic conditions. Still, the mean values of the Shannon diversity (*H*′) and Simpson's diversity (*D*) indices indicated that the HGs of BWD were characterized by moderate woody species diversity. Similarly, the woody species community structure of the HGs of the present study area was generally characterized by woody species individuals, whose numbers reduced with increasing size of individuals as measured by DBH and height, indicating the existence of selective cutting of higher individual tree species. Furthermore, the analyses of the IVI proved that *E. globulus*, *E. camaldulensis*, *R. prinoides*, *C. lusitanica*, and *C*. *macrostachyus* were the top five important woody species in HGs of the district, respectively. There were also some variations of the important woody species growing across the three agroecological zones based on the results of the IVI. These results and conclusions lead to the recommendations that (1) the local farmers of the study area should get experts' support to advance the woody species richness of their HGs via providing various multipurpose plant species, (2) other researchers should focus on assessing the local farmers' attitude and practices towards improving the management of woody species of their HGs to identify their gaps, and (3) frequent training and awareness creation campaigns should also be held to make the community well aware of the value of the HG practices for their livelihoods and environment.

## Figures and Tables

**Table 1:** Summary of the selected kebeles of BWD with total and allotted sample households.

SN. (i)	AEZ	Name of Kebele	Households per Kebele	Allotted sample size	Sampling interval (K)	Total Sample/AEZ
1	Dega	Angolela	1466	15	98	nD = 68
2	∑_*i*=1_^5^NDi = 6697	Bakelo	2159	22	98	
3		Dibut	1607	16	100	
**4**		Goshuager	542	6	90	
**5**		Wushawushign	923	9	103	

**6**	Woinadega	Goshe Bado	955	18	53	nW = 45
**7**	∑_*i*=1_^3^NWi = 2386	Mehal Amba	972	18	54	
**8**		Woiniye	459	9	51	

**9**	Kola	Kassima	1490	15	99	nK = 25
**10**	∑_*i*=1_^2^NKi = 2503	Chimbre	1013	10	101	

**Total**			11586	138		138

*Note.* AEZ = agroecological zone.

**Table 2:** Distribution of the surveyed HGs in the study area.

Agroecological zones	No. of kebeles	No. of HGs	Altitude	
			Minimum	Maximum
Dega	5	68	2483	3039
Kola	2	25	1606	1804
Woinadega	3	45	2236	2385
Overall	10	138	1606	3039

**Table 3:** List of tree and shrub species with their botanical, local, and family names as well as with their habits and voucher numbers recorded from (138) home gardens of the selected kebeles of Basona Worana district.

Botanical name	Amharic name	Family	Growth form	Voucher number
*Acacia abyssinica*	Girar	Fabaceae	S/T	AA-17
*Acacia etbaica*	Derie	Fabaceae	T	AA-33
*Acacia melanoxylon*	Omedla	Fabaceae	T	AA-40
*Allophylus abyssinicus*	Embus	Sapindaceae	S	AA-01
*Buddleia polystachya*	Anfar	Loganiaceae	S/T	AA-16
*Carissa spinarum*	Agam	Apocynaceae	S	AA-41
*Catha edulis*	Chat	Celastraceae	S	AA-19
*Chamaecytisus proliferus*	Meno	Fabaceae	S/T	AA-31
*Citrus aurantifolia*	Lomi	Rutaceae	T	AA-07
*Citrus sinensis*	Birtukan	Rutaceae	T	AA-34
*Coffea arabica*	Buna	Rubiaceae	S	AA-20
*Commiphora africana*	Anka	Burseraceae	T	AA-18
*Cordia africana*	Wanza	Boraginaceae	T	AA-39
*Croton macrostachyus*	Bisana	Euphorbiaceae	S/T	AA-09
*Cupressus lusitanica*	Yeferenj Tid	Cupressaceae	T	AA-28
*Discopodium penninervum*	Ameraro	Solanaceae	S	AA-21
*Dodonaea viscosa*	Kitkta	Sapindaceae	S	AA-29
*Dovyalis abyssinica*	Koshem	Flacourtiaceae	T	AA-03
*Ekebergia capensis*	Lol	Meliaceae	T	AA-12
*Erythrina abyssinica*	Korch	Fabaceae	T	AA-38
*Eucalyptus camaldulensis*	Key Bahirzaf	Myrtaceae	T	AA-30
*Eucalyptus globulus*	Nech Bahirzaf	Myrtaceae	S/T	AA-06
*Euclea schimperi*	Dedeho	Ebenaceae	S	AA-32
*Euphorbia abyssinica*	Kulkual	Euphorbiaceae	T	AA-13
*Euphorbia tirucalli*	Kinchib	Euphorbiaceae	T	AA-35
*Ficus sycomorus*	Bamba	Moraceae	T	AA-11
*Grewia ferruginea*	Lenqoata	Tiliaceae	S/T	AA-25
*Juniperus procera*	Yehabesha Tid	Cupressaceae	T	AA-26
*Justicia schimperiana*	Sensel	Acanthaceae	S	AA-02
*Maytenus arbutifolia*	Atat	Celastraceae	S	AA-27
*Millettia ferruginea*	Birbira	Fabaceae	T	AA-14
*Nuxia congesta*	Checho	Buddleiaceae	S	AA-08
*Olea africana*	Woyra	Oleaceae	S/T	AA-15
*Pinus patula*	Pachula	Pinaceae	T	AA-05
*Podocarpus falcatus*	Zigba	Podocarpaceae	T	AA-24
*Rhamnus prinoides*	Gesho	Rhamnaceae	S/T	AA-37
*Rhus vulgaris*	Yeregna kolo	Anacardiaceae	S	AA-10
*Ricinus communis*	Gulo	Euphorbiaceae	S/T	AA-42
*Schinus molle*	Qundo berbere	Anacardiaceae	T	AA-22
*Vernonia amygdalina*	Girawa	Asteraceae	S/T	AA-36
*Vernonia auriculifera*	Gujo	Asteraceae	S	AA-04
*Ziziphus spina-christi*	Geba/Qurqura	Rhamnaceae	T	AA-23

*Note.* T = tree, S = shrub, S/T = shrub and tree.

**Table 4:** Woody species richness of HGs in different agroecological zones and kebeles of BWD.

Agroecology	Richness	Rank	Kebeles	Richness	Rank
Dega	14	3	Angolela	11	6
			Bakelo	10	7
			Dibut	8	10
			Goshuager	9	9
			Wushawushign	10	7

Kola	33	1	Chimbre	24	1
			Kassima	21	2

Woinadega	19	2	Goshe Bado	18	3
			Mehal Amba	17	4
			Woiniye	16	5

**Table 5:** Woody species abundance of HGs in different agroecologies and kebeles of BWD.

Agroecology	Abundance	Kebeles	Abundance	Rank
Dega	3259	Angolela	715	3
		Bakelo	818	2
		Dibut	559	6
		Goshuager	194	10
		Wushawushign	973	1

Kola	606	Chimbre	270	9
		Kassima	336	8

Woinadega	1610	Goshe Bado	423	7
		Mehal Amba	566	5
		Woiniye	621	4

Overall	5475	—	5475	—

**Table 6:** Comparison of the Sorenson similarity (SS) of the woody species of the HGs between the two and of the multiple site similarity index (MSSI) among three agroecological zones of BWD.

Agroecology	SS			MSSI
	Dega	Kola	Woinadega	Dega, Woinadega, and Kola^*∗*^
Dega	1			
Kola	28%	1		
Woinadega	40%	32%	1	
Overall^*∗*^	—	—	—	36 (%)

Note. The asterisk (^*∗*^) refers to the overall similarity among HGs of the three agroecological zones.

**Table 7:** Mean values of woody species richness, abundance, diversity indices of Shannon and Simpson, and evenness of the HGs across the three agroecological zones of BWD.

Indices/variables	Mean ± SE			Overall HGs (*N* = 138)
	Dega (*n* = 68)	Woinadega (*n* = 45)	Kola (*n* = 25)	
Richness	3.21 (±1.55)	4.60 (±1.48)	5.88 (±1.59)	4.14 (±1.84)
Abundance	47.93 (±47.12)	35.78 (±32.64)	24.24 (±12.23)	39.67 (±39.2)
Shannon diversity (*H*′)	0.84 (±0.51)	1.14 (±0.31)	1.48 (±0.28)	1.05 (±0.48)
Simpson's diversity (D)	0.47 (±0.27)	0.60 (±0.13)	0.72 (±0.09)	0.55 (±0.23)
Evenness (*H*′/*H*_max_)	0.69 (±0.36)	0.78 (±0.15)	0.86 (±0.10)	0.75 (±0.27)

Note. HGs = home gardens, SE = standard error.

**Table 8:** Multiple comparisons of means of woody species richness, abundance, Shannon and Simpson diversity indices, and evenness in the HGs of BWD.

(I) Agroecology	(J) Agroecology	Mean difference (I-J)	SE	*p* value	95% CI	
					LB	UB
*Dependent variable: richness*						
Dega	Woinadega	−1.39^*∗∗*^	0.30	≤0.000	−2.09	−0.69
Dega	Kola	−2.67^*∗∗*^	0.36	≤0.000	−3.53	−1.82
Woinadega	Kola	−1.28^*∗*^	0.38	≤0.003	−2.19	−0.37

*Dependent variable: abundance*						
Dega	Woinadega	12.15	7.38	0.230	−5.35	29.64
Dega	Kola	23.69^*∗*^	8.99	0.025	2.39	44.98
Woinadega	Kola	11.54	9.58	0.453	−11.17	34.25

*Dependent variable: Shannon diversity index (H*′)						
Dega	Woinadega	−0.31^*∗*^	0.08	≤0.001	−0.50	−0.12
Dega	Kola	−0.65^*∗∗*^	0.10	≤0.000	−0.88	−0.41
Woinadega	Kola	−0.34^*∗*^	0.10	0.004	−0.59	−0.09

*Dependent variable: Simpson's diversity index (D)*						
Dega	Woinadega	−0.13^*∗*^	0.04	0.005	−0.22	−0.03
Dega	Kola	−0.25^*∗∗*^	0.05	≤0.000	−0.36	−0.13
Woinadega	Kola	−0.12^*∗*^	0.05	0.049	−0.25	−0.001

*Dependent variable: evenness (H*′*/H*_max_)						
Dega	Woinadega	−0.09	0.05	0.223	−0.21	0.04
Dega	Kola	−0.16^*∗*^	0.06	0.029	−0.31	−0.01
Woinadega	Kola	−0.08	0.07	0.494	−0.24	0.08

*Note.* ^*∗∗*^The mean difference is significant at *p* value <0.001, ^*∗*^the mean difference is significant at *p* value <0.05. CI = confidence Interval, LB = lower bound, UB = upper bound.

**Table 9:** Mean values of stand variables of woody species in HGs across agroecologies of BWD.

Stand characteristics/variables	Mean (±SE)			Overall (*N* = 138)
	Dega (*n* = 68)	Woinadega (*n* = 45)	Kola (*n* = 25)	
DBH (cm)	17.30 (±13.89)	12.92 (±8.88)	11.54 (±6.58)	14.23 (±10.90)
Height (m)	7.76 (±5.15)	5.30 (±3.85)	4.54 (±2.74)	6.04 (±4.39)
Basal area (m^2^·ha^−1^)	40.10 (±42.03)	24.86 (±37.61)	6.50 (±6.43)	29.02 (±38.53)
Crown area (m^2^)^a^	4.92 (±4.13)	4.14 (±3.69)	6.35 (±6.29)	5.00 (±4.74)

*Note.* ^a^ = the crown area was calculated for individuals of the woody species which exist as trees.

**Table 10:** Test comparisons for means of stand variables of woody species of HGs among the three agroecologies of BWD.

(I) Agroecology	(J) Agroecology	Mean difference (I-J)	SE	*p* value	95% C I	
					LB	UB
*Dependent variable: DBH*						
Dega	Woinadega	4.37^*∗∗*^	1.03	≤0.000	1.95	6.80
Dega	Kola	5.76^*∗∗*^	1.14	≤0.000	3.09	8.43
Woinadega	Kola	1.39	1.15	0.449	−1.31	4.08

*Dependent variable: height*						
Dega	Woinadega	2.46^*∗∗*^	0.41	≤0.000	1.51	3.41
Dega	Kola	3.22^*∗∗*^	0.45	≤0.000	2.17	4.26
Woinadega	Kola	0.75	0.45	0.216	−0.30	1.81

*Dependent variable: basal area*						
Dega	Woinadega	15.24	7.05	0.082	−1.50	31.91
Dega	Kola	33.60^*∗∗*^	8.58	≤0.000	13.24	53.90
Woinadega	Kola	18.36	9.15	0.114	−3.32	40.05

*Dependent variable: crown area* ^a^						
Dega	Woinadega	0.78	0.47	0.223	−0.33	1.89
Dega	Kola	−1.43^*∗*^	0.52	0.018	−2.65	−0.20
Woinadega	Kola	−2.21^*∗∗*^	0.53	≤0.000	−3.45	−0.97

Note. ^*∗∗*^The mean difference is significant at *p* value ≤0.001, but ^*∗*^ is significant at *p* value <0.05. CI = confidence Interval, SE = standard error, LB = lower bound, UB = upper bound, ^a^ = comparisons are made for trees.

**Table 11:** Relative frequency (RF), relative abundance (RA), relative dominance (RD), and importance value index (IVI) of woody species in HGs of BWD.

Botanical name	RF (%)	RA (%)	RD (%)	IVI	Rank
*Eucalyptus globulus*	13.1	35.21	45.04	93.35	1
*Eucalyptus camaldulensis*	5.40	17.41	22.23	45.04	2
*Rhamnus prinoides*	7.70	11.51	3.19	22.40	3
*Cupressus lusitanica*	6.30	5.90	10.13	22.33	4
*Croton macrostachyus*	9.80	4.37	2.77	16.94	5
*Pinus patula*	4.40	4.42	4.35	13.17	6
*Buddleia polystachya*	8.00	3.74	0.70	12.44	7
*Acacia abyssinica*	5.90	2.37	1.05	9.32	8
*Juniperus procera*	1.90	1.64	5.69	9.23	9
*Euphorbia abyssinica*	5.20	1.63	0.42	7.25	10
*Euphorbia tirucalli*	2.80	1.79	1.21	5.80	11
*Discopodium penninervum*	3.80	1.70	0.10	5.60	12
*Olea africana*	4.20	0.69	0.57	5.46	13
*Chamaecytisus proliferus*	2.80	1.83	0.31	4.94	14
*Cordia africana*	3.30	0.69	0.38	4.37	15
*Ricinus communis*	2.40	1.19	0.21	3.80	16
*Vernonia amygdalina*	2.40	0.58	0.25	3.23	17
*Acacia melanoxylon*	1.20	0.53	0.58	2.31	18
*Grewia ferruginea*	1.20	0.49	0.07	1.76	19
*Carissa spinarum*	1.00	0.57	0.014	1.58	20
*Dodonaea viscosa*	1.20	0.27	0.01	1.48	21
*Millettia ferruginea*	0.70	0.22	0.14	1.06	22
*Ekebergia capensis*	0.70	0.15	0.05	0.90	23
*Justicia schimperiana*	0.30	0.27	0.01	0.58	24
*Maytenus arbutifolia*	0.50	0.05	0.001	0.55	25
*Catha edulis*	0.30	0.18	0.004	0.48	26
*Podocarpus falcatus*	0.20	0.09	0.13	0.42	27
*Ficus sycomorus*	0.20	0.02	0.16	0.38	28
*Erythrina abyssinica*	0.30	0.04	0.02	0.36	29
*Schinus molle*	0.20	0.04	0.10	0.34	30
*Vernonia auriculifera*	0.20	0.09	0.02	0.31	31
*Citrus sinensis*	0.20	0.04	0.04	0.28	32
*Coffea arabica*	0.20	0.07	0.01	0.28	32
*Allophylus abyssinicus*	0.20	0.04	0.01	0.25	34
*Citrus aurantifolia*	0.20	0.04	0.01	0.25	34
*Ziziphus spina-christi*	0.20	0.02	0.02	0.24	36
*Acacia etbaica*	0.20	0.02	0.01	0.23	37
*Rhus vulgaris*	0.20	0.02	0.01	0.23	38
*Commiphora africana*	0.20	0.02	0.002	0.22	39
*Nuxia congesta*	0.20	0.02	0.001	0.22	39
*Dovyalis abyssinica*	0.20	0.02	0.002	0.22	39
*Euclea schimperi*	0.20	0.02	0.002	0.22	39

**Table 12:** Relative frequency (RF), relative abundance (RA), relative dominance (RD), and importance value index (IVI) of woody species of HGs in different agroecologies of BWD.

Botanical name	RF (%)	RA (%)	RD (%)	IVI	Rank
*Dega*					
*Eucalyptus globulus*	21.60	44.61	44.67	110.88	1
*Eucalyptus camaldulensis*	8.70	20.25	22.41	51.36	2
*Cupressus lusitanica*	11.90	8.10	12.09	32.09	3
*Pinus patula*	11.00	7.36	6.37	24.73	4
*Rhamnus prinoides*	10.10	8.16	3.95	22.21	5
*Juniperus procera*	4.60	2.67	8.33	15.60	6
*Buddleia polystachya*	7.80	2.42	0.44	10.66	7
*Discopodium penninervum*	7.80	2.27	0.13	10.20	8
*Ricinus communis*	6.40	1.99	0.31	8.70	9
*Acacia melanoxylon*	3.20	0.89	0.85	4.94	10

*Kola*					
*Euphorbia tirucalli*	10.9	16.17	29.95	57.02	1
*Croton macrostachyus*	15.6	14.03	13.84	43.47	2
*Cordia africana*	12.9	6.27	9.42	28.59	3
*Rhamnus prinoides*	4.8	18.81	4.04	27.65	4
*Euphorbia abyssinica*	10.9	9.08	7.58	27.56	5
*Eucalyptus globulus*	6.1	7.43	8.78	22.31	6
*Acacia abyssinica*	6.1	4.79	6.02	16.91	7
*Grewia ferruginea*	4.8	4.46	1.72	10.98	8
*Millettia ferruginea*	2.7	1.98	3.39	8.07	9
*Olea africana*	4.1	1.49	2.00	7.59	10

*Woinadega*					
*Eucalyptus globulus*	9.2	26.65	51.21	87.06	1
*Eucalyptus camaldulensis*	4.8	17.70	24.72	47.22	2
*Croton macrostachyus*	15.9	9.57	7.91	33.38	3
*Rhamnus prinoides*	7.2	15.53	1.22	23.95	4
*Acacia abyssinica*	12.1	6.27	2.88	21.25	5
*Buddleia polystachya*	12.1	7.20	1.06	20.36	6
*Cupressus lusitanica*	4.8	3.66	6.83	15.29	7
*Olea africana*	8.2	1.74	1.75	11.69	8
*Chamaecytisus proliferus*	4.8	4.72	0.76	10.28	9
*Euphorbia abyssinica*	6.3	1.99	0.36	8.65	10

## Data Availability

All data generated and analyzed during this study were included in this published article and its supplementary file.

## Supplementary Materials

Supplementary Materials Supplementary file 1: photos taken while the researcher was taking data at HGs of three kebeles of Basona Worena district (photos of (a) and (b) showing while measuring the diameters of trees at breast height and (c) showing while measuring the diameter of a tree crown).
