# Supplementary material for: Biodiversity and Structural Analysis of Woody Plant Species of Home Gardens in Basona Worana District, North Shoa Zone of Central Ethiopia
Source: Scientifica (Cairo). 2024 Feb 8;2024:5563636. doi: 10.1155/2024/5563636 (PMC10869191; doi:10.1155/2024/5563636)

Supplementary file 1: Photos taken while the researcher was taking data at HGs of three kebeles of Basona-Worena District (Photos of a) and b) showing while measuring the diameters of trees at breast height, c) showing while measuring the diameter of a tree crown)

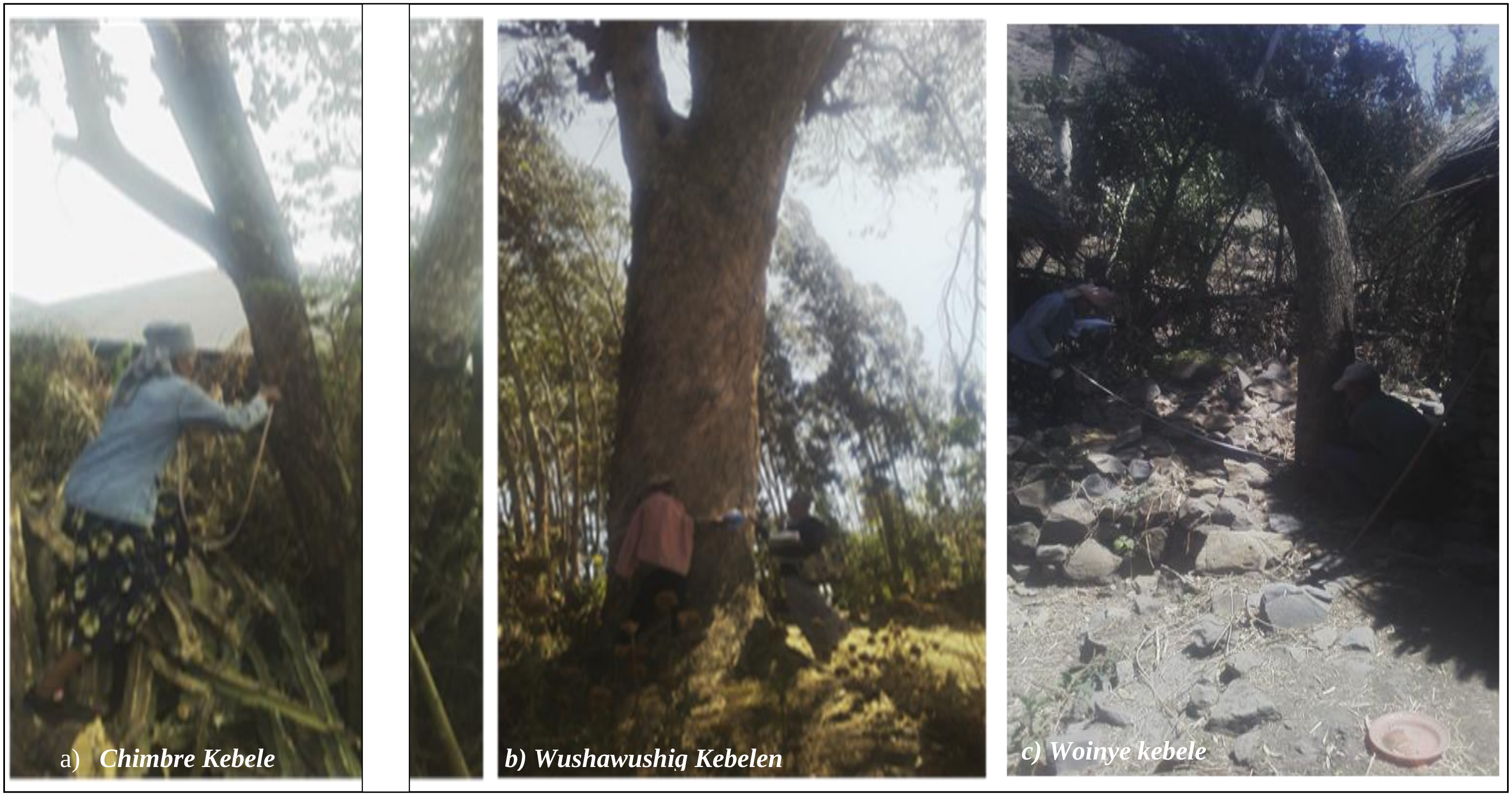

Supplement: Supplementary Materials — Supplementary file 1: photos taken while the researcher was taking data at HGs of three kebeles of Basona Worena district (photos of (a) and (b) showing while measuring the diameters of trees at breast height and (c) showing while measuring the diameter of a tree crown). [file 5563636.f1.pdf]
